# Self-perceived mental health related to coping strategies among studentsat a private university in Colombia

**DOI:** 10.15649/cuidarte.5315

**Published:** 2026-10-16

**Authors:** Etilvia Leonor Campo-Torregroza, Kisi Nayidis Padilla-Constante, Greys Patricia Castro-Melo

**Affiliations:** 1 Universidad Cooperativa de Colombia, Santa Marta, Colombia. E-mail: etilvia.campo@campusucc.edu.co Universidad Cooperativa de Colombia Santa Marta Colombia etilvia.campo@campusucc.edu.co; 2 Universidad Cooperativa de Colombia, Santa Marta, Colombia. E-mail: kisi.padilla@campusucc.edu.co Universidad Cooperativa de Colombia Santa Marta Colombia kisi.padilla@campusucc.edu.co; 3 Universidad Cooperativa de Colombia, Santa Marta, Colombia. E-mail: greys.castro@campusucc.edu.co Universidad Cooperativa de Colombia Santa Marta Colombia greys.castro@campusucc.edu.co

**Keywords:** Students, Higher Education, Mental Health, Coping, Estudiantes, Educación Superior, Salud Mental, Afrontamiento, Estudantes, Ensino Superior, Saúde Mental, Enfrentamento

## Abstract

**Introduction:**

According to the Colombian Association of Universities (ASCUN), 40% of university students in Colombia experience symptoms of anxiety and depression, reflecting mental health disorders.

**Objective:**

To identify the relationship between self-perceived mental health and coping strategies among university students.

**Materials and Methods:**

This quantitative, cross-sectional, correlational study included a population of 4,107 students. A proportionate stratified random sampling approach was used to select 352 students from nine undergraduate programs at a private university in Colombia. The 12-item General Health Questionnaire (GHQ-12), supplemented with items assessing sociodemographic characteristics, and the Fernández-Abascal Coping Styles and Stress Coping Strategies Scale were used. Statistical analysis and data processing were performed using Stata 16.0.

**Results:**

Self-perceived mental health problems were more prevalent among female students, and the related coping strategies were: (1) problem solving, (2) seeking social support, (3) waiting, (5) emotional avoidance, (6) aggressive reaction, (9) positive reappraisal, (10) expression of coping difficulty, (11) denial, and (12) autonomy.

**Discussion:**

In contrast to previous studies reporting a higher prevalence of students with mental health problems, the prevalence observed in the present study was lower.

**Conclusion:**

The coping strategies used by university students may determine their self-perceived mental health problems.

## Resumen

**Introducción:**

Según la Asociación Colombiana de universidades (ASCUN), el 40% de los estudiantes universitarios de Colombia presentan síntomas de ansiedad y depresión, alteraciones estas de su salud mental.

**Objetivo:**

Identificar la relación entre la autopercepción de salud mental y las estrategias de afrontamiento en el estudiante universitario.

**Materiales y métodos:**

Estudio cuantitativo, correlacional de corte transversal. Población de 4.107, muestreo estratificado porcentual por selección aleatoria de 352 estudiantes de nueve programas de pregrado de una universidad privada de Colombia. Los instrumentos utilizados fueron; la escala de la salud general (GHQ-12) al que se le incorporaron ítems para los datos sociodemográficos; relacionándose con los resultados de la Escala de estilos y estrategias de afrontamiento al estrés de Fernández-Abascal. Para la analítica y tratamiento de los datos se realizaron procedimientos estadísticos, con Stata 16.0.

**Resultados:**

La autopercepción de alteración en la salud mental fue mayor en el sexo femenino y las estrategias de afrontamiento relacionadas fueron: (1) solución de problema, (2) búsqueda de apoyo social, (3) espera, (5) evitación emocional, (6) reacción agresiva, (9) revaluación positiva, (10) expresión de dificultad de afrontamiento, (11) negación y (12) autonomía.

**Discusión:**

A diferencia de otros estudios que reportan mayor frecuencia de estudiantes con alteraciones en su salud mental, en el estudio actual fue menor este porcentaje.

**Conclusiones:**

La utilización de las estrategias de afrontamiento determinarán la alteración de la salud mental autopercibida del universitario.

## Resumo

**Introdução:**

De acordo com a Associação Colombiana de Universidades (ASCUN), 40% dos estudantes universitários na Colômbia apresentam sintomas de ansiedade e depressão, transtornos mentais.

**Objetivo:**

Identificar a relação entre a saúde mental autopercebida e as estratégias de enfrentamento em estudantes universitários.

**Materiais e métodos:**

Foi realizado um estudo transversal, correlacional e quantitativo. A população foi composta por 4.107 estudantes, dos quais 352 estudantes de nove cursos de graduação de uma universidade privada na Colômbia foram selecionados aleatoriamente por meio de amostragem estratificada percentual. Os instrumentos utilizados foram o Questionário de Saúde Geral (GHQ-12), que incluiu itens para dados sociodemográficos, e os resultados da Escala de Estilos e Estratégias de Enfrentamento de Fernández-Abascal. Os procedimentos estatísticos foram realizados utilizando o STATA 16.0 para análise e processamento dos dados.

**Resultados:**

A autopercepção de saúde mental prejudicada foi maior em mulheres, e as estratégias de enfrentamento utilizadas foram: (1) resolução de problemas, (2) busca de apoio social, (3) espera, (5) evitação emocional, (6) reação agressiva, (9) reavaliação positiva, (10) expressão de dificuldade de enfrentamento, (11) negação e (12) autonomia.

**Discussão:**

Ao contrário de outros estudos que relatam uma frequência maior de estudantes com saúde mental prejudicada, essa porcentagem foi menor no presente estudo.

**Conclusões:**

O uso de estratégias de enfrentamento determinará o grau de comprometimento da saúde mental em estudantes universitários.

## Introduction

The World Health Organization (WHO) defines mental health as a state of well-being in which individuals realize their abilities, cope with the normal stresses of life, work productively, and contribute to their community. From a positive perspective, mental health constitutes the foundation of individual well-being and the effective development of communities^1^. Law 1616 of 2013 defines mental health as a dynamic state that is reflected in individuals’ daily lives through the use of emotional resources, changes in cognitive capacity, and the transformation of thought processes, enabling them to meet challenges and live within their communities^2^. According to Joyce Travelbee, mental health is not something an individual possesses at a particular moment; rather, it reflects who that individual is at a specific stage, in a specific setting, in a specific sociocultural status, and at specific time. For Travelbee, a person is defined by a set of human qualities and capacities, including the ability to love, cope with reality, and find meaning and purpose in life^3^,^4^.

According to the WHO, self-perceived mental health problems are characterized by changes in an individual’s thinking processes, emotional regulation, or behavioral patterns, and are associated with distress or impairment in functional areas. Anxiety and depression are the most common disorders and are among the conditions for which university students, particularly those in late adolescence, most frequently seek care^5^,^6^.

University students are considered a vulnerable population because of their developmental stage and age, as 75% of mental disorders have their onset before the age of 25, and the highest mortality rates occur between 15 and 24 years of age^7^-^11^. Some studies have reported that individuals in this age group are particularly vulnerable because they face stress-inducing situations within the university environment that negatively affect mental health. During this period, the adjustments, challenges, and changes they experience may have either positive or negative effects. Sleep disturbances, the initiation of romantic relationships, and the potential use and misuse of alcohol and drugs may contribute to psychological problems^12^-^15^. Likewise, certain personality traits may increase an individual’s susceptibility to developing mental health problems^7^,^8^.

Given the high vulnerability of the global population resulting from diverse global situations and dynamics, the World Health Organization established recommendations for all countries through the Comprehensive Mental Health Action Plan 2013-2030, urging the implementation of strategies aimed at strengthening mental health across populations, reducing risk factors, and increasing investment in the financial resources required to address mental health^16^.

Therefore, in Colombia, different policies have been adopted to address these requirements by developing strategic plans aimed at strengthening the population’s mental health. These policies include Law 1616 of 2013, which established the Mental Health Law^2^, and the Ten-Year Public Health Plan 2012-2021, in which mental health was prioritized as one of the nation’s strategic pillars in public health^17^,^18^.

According to Frydenberg et al.^19^, coping refers to behavioral and cognitive strategies used to achieve effective transition and adaptation. In problematic situations generated by the stress experienced by an individual, cognitive or behavioral actions may become evident, including thoughts and feelings that allow the individual to avoid or reduce the impact of such situations, either by modifying the situation itself or by changing its meaning, thereby reducing the degree of threat or the symptoms of stress^20^.

Coping strategies act as protective factors for mental health. The use of functional or productive strategies is associated with quality of life, well-being, adaptation, and adjustment to the university environment^8^,^21^,^22^. Coping strategies are grouped into three forms that reflect favorable behavioral aspects, which promote overcoming the stressful event, and unfavorable behavioral aspects, which not only hinder the resolution of the stressful situation but also place emotional stability at risk.

These forms of coping may include strategies aimed at solving the problem individually (problem solving, positive reappraisal, autonomy), strategies based on seeking support (seeking social support, seeking professional support, religion), and unproductive strategies (waiting, emotional avoidance, aggressive reaction, expression of coping difficulties, denial)^23^. Accordingly, is there a relationship between self-perceived mental health and coping strategies among university students?.

## Materials and Methods

**Study design and population**

This was a quantitative, descriptive, cross-sectional correlational study. The population consisted of 4,107 students from nine undergraduate programs at a private Colombian university^24^,^25^. The sample was calculated using proportionate stratified sampling, with 352 students randomly selected at a 99% confidence level, 3% margin of error, and an expected proportion of 5%. Participants were contacted by the researchers on the university campus during the second semester of 2022 and the first semester of 2023. After signing the informed consent form, they completed the instruments themselves with guided support.

Inclusion criteria: enrolled students aged 18 to 25 years who participated voluntarily. Students belonging to Indigenous populations, those diagnosed with and receiving treatment for mental illness, and those with psychoactive substance abuse were excluded.

**Variables and data collection**

The independent variable was assessed using the Goldberg General Health Questionnaire-12 (GHQ-12), which was validated in Colombia by Ruiz FJ, García-Beltrán DM, and Suárez-Falcón JC, with a Cronbach’s alpha of 0.90. This 12-item scale uses a 4-point Likert-type response format, where 0 = more than usual, 1 = same as usual, 2 = less than usual, and 3 = much less than usual. The total score allows the identification of self-perceived mental health problems. The response variable, self-perceived health status, comprised two categories: (1) no self-perceived mental health problems (0–11 points) and (2) self-perceived mental health problems (12–36 points), based on the summed score of the items scored from 0 to 3^26^-^28^. Items were added to the scale header to collect demographic data, including age, sex, marital status, socioeconomic stratum, living arrangement, place of origin, economic activity, undergraduate program, and semester.

The coping mechanisms used were identified using the Fernández-Abascal Coping Styles and Strategies for Stress Scale (EEC), modified and validated in Colombia by Londoño et al.^29^, with an overall Cronbach’s alpha of 0.85 and an explained variance of 62.04%. The Modified Coping Strategies Questionnaire (EEC-M) consists of 69 items divided into 12 factors, each with a Cronbach’s alpha greater than 0.70. The factors are listed as follows: (1) PS, problem solving; (2) SSS, seeking social support; (3) W, waiting; (4) R, religion; (5) EA, emotional avoidance; (6) SPS, seeking professional support; (7) AR, aggressive reaction; (8) CA, cognitive avoidance; (9) PR, positive reappraisal; (10) ECD, expression of coping difficulty; (11) D, denial; and (12) A, autonomy. Each item comprising the factors uses a Likert-type response option, with frequency scores ranging from 1 to 6: never, almost never, sometimes, often, almost always, and always.

**Data analysis**

Once the data had been collected and organized in an Excel spreadsheet, analyses were conducted using descriptive, inferential, and multivariable statistics to identify the coping styles most frequently used by students who self-perceived their mental health as having problems, compared with those who perceived it as having no problems.

For each coping style and strategy, the percentage of use was calculated by considering the maximum score obtained by multiplying the number of items by the maximum possible score (6 points). Sociodemographic characteristics were also included, such as age, sex, living arrangement, socioeconomic stratum, place of origin, academic program, and occupation.

Numerical variables are presented as medians and quartiles because they did not show a normal distribution; categorical variables are expressed as absolute frequencies and percentages.

In the inferential analysis, variables were selected based on their theoretical relevance, and some sociodemographic characteristics were recategorized to improve model fit. Chi-square and Wilcoxon tests were applied, with a significance level of 5% (α = 0.05).

Finally, a logistic regression model, adjusted for sociodemographic variables, was developed to address the research question. Analyses were performed using Stata 16.0. The complete data are freely available in Mendeley Data^30^.

**Ethical considerations**

The study complied with ethical standards, respecting voluntary participation through the acceptance and signing of the informed consent form, and ensuring confidentiality and anonymity by assigning a numerical code to the instruments, which were kept in the researchers’ custody. The research began after approval by the Institutional Bioethics Committee of Universidad Cooperativa de Colombia, Minutes No. 008, dated November 11, 2020. International and national ethical regulations were considered, including the updated version of the Declaration of Helsinki and Resolution 8430 of 1993 of the Colombian Ministry of Health. The study was classified as risk-free because it did not involve interventions that altered participants’ variables or the intentional modification of biological, physiological, or psychological variables; participation was limited to the completion of instruments administered by the researchers^31^,^32^.

## Results

The sample consisted of 352 students, of whom 61.65% (n=217) were female, and 38.25% (n=135) were male; the median age was 20 years. Most participants were medical students 22.44% (n=79); and nursing students 20.74%; (n=73). A total of 94.89% (n=334) reported being single; more than 80% (n=217) belonged to the first three socioeconomic strata; 92.33% (n=325) reported being students as their occupation; and 59.66% (n=210) lived with their parents. Finally, the main place of origin was Santa Marta 57.67% (n=203) Table 1.

**Table 1:** Sociodemographic characteristics of the sample (n=352)

Variable	Total % (n)
Age - Md [IQR]	20 [19 ; 22]
Sex	
Male	38.25 (135)
Female	61.65 (217)
Academic program	
Medicine	22.44 (79)
Nursing	20.74 (73)
Law	11.65 (41)
Business administration	5.68 (20)
Commerce	3.41 (12)
Accounting	7.75 (28)
Electrical Engineering	2.56(9)
Civil Engineering	8.81 (31)
Psychology	59 (16.76)
Marital status	
Single	94.89 (334)
Married	1.14 (4)
Common-law union	3.69 (13)
Divorced/separated	0.0 (0)
Widowed	0.28 (1)
Socioeconomic stratum	
1	20.17 (71)
2	26.14 (2)
3	40.91 (144)
4	9.94 (35)
5	1.14 (4)
6	1.7 (6)
Occupation	
Student	92.33(325)
Employee/student	7.67 (27)
Living arrangement	
Parents	59.66 (210)
Siblings	5.4 (19)
Uncles/aunts	4.26 (15)
Grandparents	4.83 (17)
Spouse	1.99 (7)
Boarding house resident	19.6 (69)
Alone	4.26 (15)
Place of origin	
Santa Marta	57.67 (203)
Other	42.33 (149)

n: sample; Md: median; IQR: interquartile range

Regarding coping styles or strategies and self-perceived mental health status, Factors (1) problem solving and (9) positive reappraisal showed the highest percentages of use in the study sample. In contrast, the least frequently used factors were (6) seeking professional support and (7) aggressive reaction. With respect to health status, 53.41% (n=188) obtained scores corresponding to the category of self-perceived mental health problems Table 2.

**Table 2:** Coping styles and health status according to the GHQ-12 (n = 352)

Variable	Total Md [IQR]
Factor 1: PS – Problem solving	57.40 [48.15; 66.66]
Factor 2: SSS – Seeking social support	47.62 [38.09; 59.52]
Factor 3: W – Waiting	44.44 [35.18; 55.55]
Factor 4: R – Religion	52.38 [39.28; 69.05]
Factor 5: EA – Emotional avoidance	56.25 [43.75; 68.75]
Factor 6: SPS – Seeking professional support	33.33 [16.67; 50.00]
Factor 7: AR – Aggressive reaction	40.00 [30.00; 55.00]
Factor 8: CA – Cognitive avoidance	53.33 [46.67; 63.33]
Factor 9: PR – Positive reappraisal	56.67 [46.67; 70.00]
Factor 10: ECD – Expression of coping difficulty	50.00 [37.50; 58.33]
Factor 11: D – Denial	50.00 [38.89; 58.33]
Factor 12: A – Autonomy	50.00 [41.67; 66.67]
Perceived mental health - % (n)	
No self-perceived mental health problems	46.59 (164)
Self-perceived mental health problems	53.41 (188)

n: sample; Md: median; IQR: interquartile range

Table 3 shows the factors associated with health status. Among the factors statistically significantly associated with self-perceived mental health problems were female sex and coping factors or strategies 1, 2, 3, 5, 7, 9, 10, 11, and 12. Overall, higher scores for self-perceived mental health problems were observed among individuals who used coping strategies 3, 5, 7, 10, 11, and 12. In contrast, individuals with no self-perceived mental health problems showed higher utilization scores for Factors 1, 2, and 9.

Finally, Table 4 presents the multivariable model adjusted for sociodemographic variables. Greater use of Factor 1 was found to be a protective factor for self-perceived mental health problems, compared with those who did not present such problems. In contrast, Factor 10 was identified as a risk factor for self-perceived mental health problems, compared with those who did not present such problems. These estimates were adjusted for age, sex, living arrangement, occupation, place of origin, and the remaining factors included in the model.

**Table 3:** Factors associated with self-perceived health status (n=352)

Variable	No self-perceived mental health problems (n=164) % (n)	Self-perceived mental health problems (n=188) % (n)	p-value
Age-Md [IQR]	20 [19 ; 22]	20 [19 ; 22]	0.504*
Sex			<0.0001**
Male	48.78 (80)	29.26 (55)	
Female	51.22 (84)	70.74 (133)	
Marital status			0.646**
Single/Widowed	94.68(157)	94.68 (178)	
Married/common-law union	4.27 (7)	5.32 (10)	
Occupation			0.816**
Student	92.68 (152)	92.02 (173)	
Employee/student	7.32(12)	7.98 (15)	
Living arrangement			0.114**
With someone	97.56 (160)	94.15 (177)	
Alone	2.44 (4)	5.85 (11)	
Place of origin			0.109**
Santa Marta	62.20 (102)	53.72 (101)	
Other	37.80 (62)	46.28 (87)	
Factor 1: Problem solving-Md [IQR]	61.11 [51.85; 72.22]	55.55 [46.29; 62.96]	<0.0001*
Factor 2: Seeking social support-Md [IQR]	50.00 [40.47; 61.90]	45.24 [35.71; 57.14]	0.013*
Factor 3: Waiting-Md [IQR]	42.59 [32.41; 55.55]	46.29 [38.89; 57.41]	0.006*
Factor 4: Religion-Md [IQR]	52.38 [36.90; 71.43]	52.38 [40.48; 69.05]	0.961*
Factor 5: Emotional avoidance-Md [IQR]	52.08 [41.67; 64.58]	60.42 [47.92; 72.91]	<0.0001*
Factor 6: Seeking professional support-Md [IQR]	33.33 [23.33; 50.00]	33.33 [20.00; 50.00]	0.957*
Factor 7: Aggressive reaction-Md [IQR]	33.33 [23.33; 50.00]	43.33 [35.00; 60.00]	<0.0001*
Factor 8: Cognitive avoidance-Md [IQR]	53.33 [46.67; 66.67]	53.33 [43.33; 63.33]	0.298*
Factor 9: Positive reappraisal-Md [IQR]	60.00 [50.00; 76.66]	53.33 [43.33; 63.33]	<0.0001*
Factor 10: Expression of coping difficulty-Md [IQR]	45.83 [37.50; 54.17]	54.17 [45.83; 62.50]	<0.0001*
Factor 11: Denial-Md [IQR]	44.44 [38.89; 55.55]	50.00 [44.44; 61.11]	0.003*
Factor 12: Autonomy-Md [IQR]	50.00 [33.33; 66.67]	50.00 [41.67; 66.67]	0.049*

n: muestra; Me: mediana; RIQ: rango intercuartílico; *Wilcoxon test; **Chi-square test

**Table 4:** Logistic regression model for identifying factors associated with mental health problems (n = 352)

Variable	Adjusted odds ratio* (95% CI)
Factor 1: PS - Problem solving	0.967 (0.944 - 0.991)
Factor 2: SSS - Seeking social support	1.006 (0.988 - 1.023)
Factor 3: W – Waiting	0.994 (0.975 - 1.013)
Factor 5: EA - Emotional avoidance	1.010 (0.989 - 1.032)
Factor 7: AR - Aggressive reaction	1.013 (0.997 - 1.029)
Factor 9: PR - Positive reappraisal	0.986 (0.966 - 1.006)
Factor 10: ECD - Expression of coping difficulty	1.037 (1.012 - 1.062)
Factor 11: D – Denial	1.010 (0.992 - 1.029)
Factor 12: A – Autonomy	1.004 (0.989 - 1.018)

*Adjusted for age, sex, living with someone, occupation, place of origin, and the factors included in the model. Adjusted odds ratio test 8

## Discussion

University students may self-perceive mental health problems when they have access to an instrument that describes behaviors they can recognize in their everyday behavior, as is the case with the General Health Questionnaire-12 (GHQ-12). This self-perception is important because it enables students, faculty, and university bodies to intervene in these behaviors early and in a timely manner, preventing more severe disorders that may affect students’ academic performance and social coexistence^7^,^33^. Likewise, strategies that promote time management should be incorporated into student well-being plans to avoid academic overload and reduce stress.

However, published findings indicate that this self-perception is not entirely reliable because it does not identify the magnitude of the problem^34^. Conversely, reduced emotional regulation has been described among students, which limits the use of appropriate coping strategies or skills that help prevent mental health problems^33^.

The results of this study identified a substantial percentage of self-perceived mental health problems, with a lower percentage of students presenting behaviors indicative of such problems. This situation warrants special attention for timely and effective intervention and prevention^35^. Similarly, Cardona-Arias et al.^7^ reported higher frequencies of students with mental health problems, 58%, 61.92%, and 65%, compared with 53.41% (n=188) in the present study.

Regarding sex, female students showed a higher prevalence of self-perceived mental health problems, at 70.74% (n = 133). A similar result was reported by Tiga-Loza et al.^28^, in which 87.17% of the female population perceived mental health problems, showing a significant difference compared with the findings of Cardona-Arias et al.^7^, who reported 59.6%. However, another study reported no significant differences in perceptions of moderate and severe stress between sexs^36^,^37^. These findings reflect the need to address mental health problems in educational institutions through a differential approach to provide more effective responses.

Consequently, this self-perception of mental health is related to the strategies university students use. Therefore, as a statistical contribution of the study, a logistic regression model was fitted Table 4, identifying two factors in the sample with self-perceived mental health problems: Factor 1 (problem solving), which behaved as a protective factor, and Factor 10 (expression of coping difficulty), which behaved as a risk factor.

Understanding self-perceived mental health without problems as equivalent to psychological well-being, the coping strategies related to self-perception without problems were problem solving, seeking social support, and positive reappraisal. These factors, among others, were also reported as statistically significant factors related to psychological well-being^19^ by Narváez et al.^38^ among university students.

The coping strategies expression of coping difficulty, denial, and autonomy were identified as being related to the different dimensions of psychological well-being^38^. Similarly, the use of these factors was observed in the study sample. At the same time, factors such as religion and cognitive avoidance were used less frequently, and seeking professional support showed the same pattern in both studies^38^.

In the results of the present study, women who self-perceived their mental health as having problems used, among other coping strategies, avoidance and denial, and did not use seeking social support. A similar finding regarding the use of coping strategies was reported by Cuamba and Zazueta^22^ among university students and by Uribe Urzola et al.^23^ Among men, the most frequently adopted coping strategies included avoidance and denial, among others, with lower use of strategies involving seeking social support. Therefore, passive practices become evident, in which individuals believe that solutions occur without taking any action, as do avoidant practices, in which they evade situations that may generate conflict, leading to psychological consequences.

## Conclusions

Self-perceived mental health problems are related to unfavorable coping strategies, such as emotional avoidance, expression of coping difficulty, and denial. In contrast, self-perceived mental health without problems is associated with problem-solving, positive reappraisal, and seeking social support, respectively.

The study findings regarding the use of coping strategies indicate that these strategies are related to self-perceived mental health, with problem-solving acting as a protective factor, whereas expressing coping difficulty tends to be a risk factor.

The promotion of spaces within the university context is suggested, with a focus on creating programs aligned with national and international policies for mental health promotion that facilitate emotional expression and strengthen skills in using favorable coping strategies.
